# Weakly-Supervised Detection of Facial Motor Tics in Video Data Using Score-Driven Top-*k* Multiple Instance Learning

**DOI:** 10.1007/s41666-026-00233-z

**Published:** 2026-07-30

**Authors:** Nele Sophie Brügge, Frédéric Li, Ronja Schappert, Gesine Marie Sallandt, Christian Beste, Sebastian Fudickar, Marcin Grzegorzek, Alexander Münchau, Heinz Handels

**Affiliations:** 1 German Research Center for Artificial Intelligence, Lübeck, Germany; 2 Institute of Systems Motor Science, University of Luebeck, Lübeck, Germany; 3 Department of Neurology, University Hospital Medical Center Schleswig-Holstein, Lübeck, Germany; 4 Cognitive Neurophysiology, Department of Child and Adolescent Psychiatry, Faculty of Medicine, TU Dresden, Dresden, Germany; 5 Cognitive Psychology, Faculty of Psychology, Shandong Normal University, Jinan, China; 6 Fraunhofer Research Institution for Individualized and Cell-Based Medical Engineering (IMTE), Lübeck, Germany; 7 Section for Clinical Research IT (SKFIT), Institute of Medical Biometry and Statistics, University of Luebeck, Lübeck, Germany; 8 Institute of Medical Informatics, University of Luebeck, Lübeck, Germany

**Keywords:** Multiple instance learning, Video analysis, Gilles de la Tourette syndrome, Tic detection, Weak supervision, Deep learning

## Abstract

In medicine, the laborious annotation process poses a significant limitation to the creation of large labelled datasets, a challenge that is particularly pronounced for medical video data, where numerous frames require expert annotation. Weakly supervised approaches, such as Multiple Instance Learning (MIL), offer a promising solution by leveraging only video-level labels rather than frame-by-frame annotations. In this work, we tailor a top-k MIL-based approach to medical video data to detect and localise facial motor tics in videos of patients diagnosed with Gilles de la Tourette Syndrome (GTS). Our adaptations include a score-driven top-k instance selection strategy that accounts for variability in tic occurrence between subjects. This enables the effective exploitation of large unlabelled datasets by incorporating weak labels where available. Evaluated against state-of-the-art MIL models, the proposed approach achieves the highest instance-level tic detection performance, with an area under the curve of 86.73 %. These results demonstrate that facial motor tics in videos of individuals with GTS can be effectively localised using only weak video-level annotations, highlighting the potential of the proposed approach for future clinical applications.

## Introduction

Gilles de la Tourette syndrome (GTS) is a complex neurodevelopmental disorder characterised by the presence of motor and vocal tics. The condition can lead to significant social and functional impairment [1, 2]. Treatment for GTS patients includes medication and behavioural therapy to help patients recognise tics and develop strategies for controlling them [3–6]. However, the limited availability of therapists [7] and the problems associated with conducting follow-up appointments affect the effectiveness of this approach.

To address these challenges and to assist clinicians in diagnosis and treatment decisions, there has been an increasing number of studies applying machine learning to GTS, in particular for the problem of automated tic detection [8–13]. However, a common limitation of most work is the dependence on accurately labelled datasets, as conventional supervised models typically cannot be trained with noisily or weakly labelled data. Especially in medical applications, the process of labelling data requires the expertise of professionals, is laborious, and time-consuming, while accurate and reliable results are paramount. Therefore, streamlining the process of video labelling and developing unsupervised or weakly-supervised methods could enhance the efficiency and accuracy of automated tic detection.

In this work, we investigate the second-wise classification of facial motor tics in videos of patients diagnosed with GTS, using only weak video-level annotations such as the information of whether the patient is healthy or affected by GTS. We propose the use of Multiple Instance Learning (MIL) for analysing medical video data to tap into the vast reservoir of unlabelled medical data, and to make more efficient use of available resources. MIL significantly reduces the burden of human annotation by requiring only video-level labels for training models that are able to provide detailed second-wise predictions. We adopt an approach inspired by the robust temporal feature magnitude learning (RTFM) method from [14], which has been applied in the context of anomaly detection in surveillance videos. In RTFM, a classifier is trained using the *k* instances with the highest feature magnitudes. The underlying assumption is that the mean feature magnitude of abnormal snippets is higher than that of normal snippets. The method promotes large margins between the *k* snippet features with the maximum magnitudes from abnormal and normal videos. This contributes to robust and accurate classification, even when dealing with weakly or noisily labelled data.

We propose to adapt this MIL approach to a medical video dataset. There are several differences between medical video and surveillance camera or street scene data used in [14–24] (ShanghaiTech [25, 26], UCF-Crime [21], XD-Violence [27] and UCSD-Peds [28]). Standard feature extractors such as the 3D Convolutional Network (C3D) [29] or Inflated 3D Convolutional Network (I3D) [30] have limited applicability to facial video data because they were trained on very dissimilar datasets. Additionally, in medical applications, the number of abnormal snippets may vary significantly between individuals, depending on the specific disease or abnormality being targeted. For instance, in the context of GTS, the probability of tic occurrence varies greatly from one subject to another. Accounting for such differences enables using a greater number of abnormal snippets in training the MIL classifier, a modification proving particularly advantageous when working with limited training data from only few individuals.

**Fig. 1:**
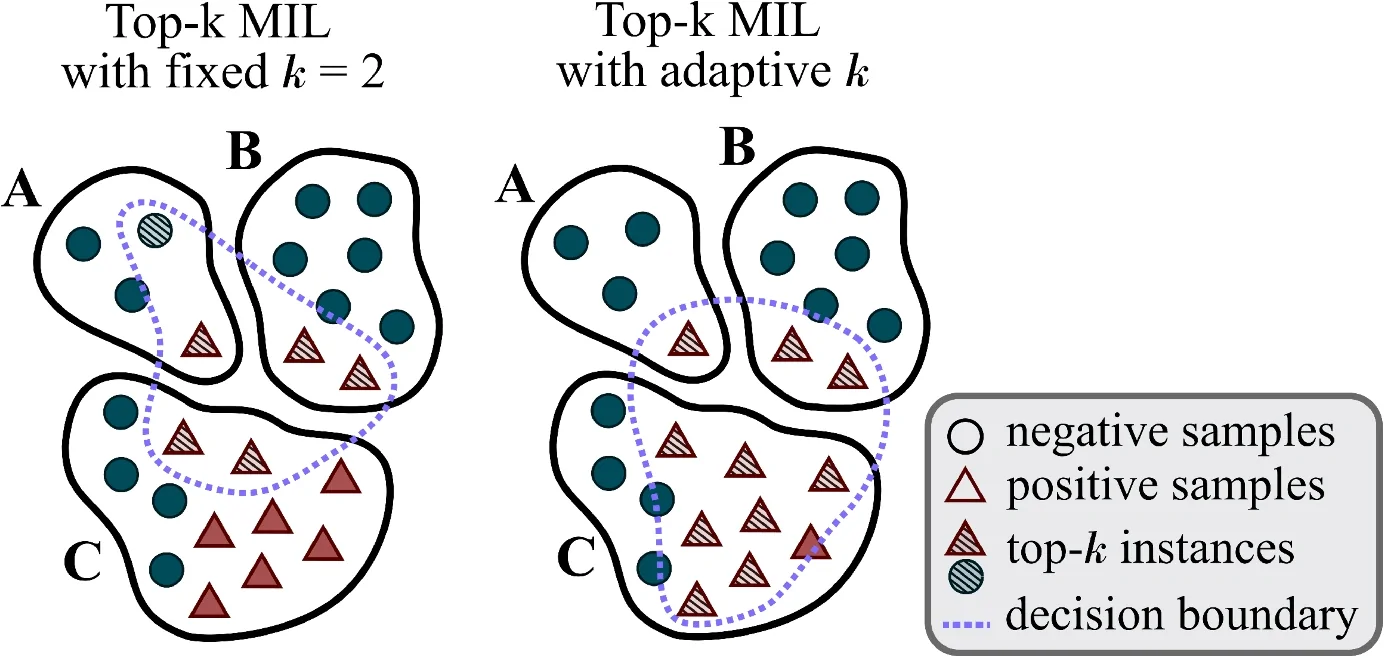
Example classification boundaries in MIL for cases where the total number of positive instances in each positive bag varies greatly. Solid lines delineate three different positive bags (**A**, **B** and **C**), each containing a different number of positive samples (red triangles). Dashed lines show a sample classification decision boundary for top-*k* MIL with fixed *k* and top-*k* MIL with *k* adapted depending on a weak label. Negative bags are not depicted for simplicity

We propose two key contributions. The first consists of modifying top-*k* MIL to account for the peculiarities of facial video data for tic detection. This is done by applying contrastive learning to facial video data. In this initial unsupervised training phase, the feature network learns to extract meaningful features from facial movements and expressions. For the MIL training phase, the input videos are cut into shorter video sequences to increase the amount of training data. These sequences are then divided into negative (healthy control (HC) subjects) and positive (patients diagnosed with GTS) bags. Our second key modification is to automatically adapt the parameter *k* depending on an associated weak label. This addresses a limitation of conventional MIL, where only a small subset of training snippets contributes to learning – even when a video contains numerous relevant events – thus underutilising the available weakly labelled data. This scenario is shown in the left part of Fig. 1 in bag C. Conversely, if there are fewer than *k* positive snippets in a positive bag, there may be a large number of snippets falsely labelled as positive, resulting in noisy labels (see bag A in Fig. 1). In response to this challenge, we determine per subject how to optimally and automatically choose *k* in the top-*k* MIL approach depending on a subject-level tic score. In this way, we aim to make the best use of the available data and to minimise the selected false-positive instances during top-*k* MIL training.

We apply our proposed method to a large dataset comprising weakly-labelled videos of both patients and HC subjects. In tic detection, it is particularly important to include data from many individuals in the training of machine learning models, given that the expression of the disorder varies substantially between patients [31, 32] and even overlaps with normal functioning [33]. Leveraging the unlabelled dataset increases the diversity of the data and thus the generalisation capabilities of the trained models, making cross-subject application feasible and more robust.

To summarise, the contributions of this paper consist of:

- We are the first to apply a MIL approach based on RTFM on medical video data, in a context of Tourette tic detection.
- We propose to use contrastive learning features that put facial movements and expressions in the foreground.
- We automatically fine-tune the parameter *k* in top-*k* MIL based on information from subject-level tic scores to address the variability in tic occurrence among individuals, ensuring that the model efficiently utilises available data for training.

In the following section, we provide a brief overview of GTS, followed by a review of related work on automated tic detection and MIL in medical applications in Section 3. We present both our densely- and weakly-labelled dataset in Section 4. Building on this, we detail our feature extraction and MIL methods in Section 5. Section 6 outlines our experimental setup, and in Section 7, we present and discuss our findings. Finally, Section 9 provides a conclusion.

## Gilles de la Tourette Syndrome

GTS is a complex neurodevelopmental disorder characterised by involuntary motor and vocal tics [34]. Tics are sudden, repetitive, non-rhythmic movements or vocalisations that range from simple, such as eye blinking or throat clearing, to more complex, like facial grimacing or coprolalia (involuntary swearing) [35–38]. A few of these manifestations are listed in Table 3 in the Appendix. The syndrome manifests in childhood or adolescence and sometimes continues into adulthood [34]. The estimated prevalence of GTS is *0.3%* to *0.9%* [39]. The condition varies in tic frequency and severity [31, 32] and can lead to significant social and functional impairments [1]. Although there is currently no cure for GTS, a multidisciplinary approach involving behavioural therapies, medications, and supportive interventions can help to manage symptoms and improve the quality of life for affected individuals [3–6]. Understanding the underlying mechanisms of GTS remains a crucial area of research to develop targeted interventions. Since there is no specific confirmatory laboratory, imaging, or genetic test, accurate tic detection relies on clinical expertise [40].

## Related Work

### Tic Detection in GTS Patient Data

There is limited prior work on the detection of tics from sensor or video data of patients diagnosed with GTS. However, this existing research often remains limited, is not representative, or does not use the potential of large unlabelled datasets.

Some works rely on one-dimensional sensor data, such as the accelerometer-based approach proposed by Barua et al. [8]. This approach exclusively focuses on data from healthy participants who are mimicking tics, potentially missing tic characteristics specific to patients with GTS. Bernabei et al. [41] also employed accelerometer data attached to the GTS patients’ trunks. Yet, this approach cannot detect facial tics due to the sensor location. In the work of Cernera et al. [11], electromyography data were gathered in addition to accelerometer data during video recordings of voluntary movements. They trained patient-specific Support Vector Machines (SVMs) to classify tic and non-tic phases. However, we use a more difficult subject-independent setting in our study, making a direct comparison of these results to ours difficult. Additionally, Shute et al. [13] used invasive deep brain stimulation electrodes to measure thalamocortical network activity signals and classified these using an SVM.

Other studies have explored the use of video recordings of GTS patients as an alternative to sensor-based approaches. The advantage of using video data lies in its independence from additional sensors and their precise positioning, not only facilitating and accelerating the data acquisition process but also making it more comfortable for the patient. Tang et al. [12] addressed tic detection in untrimmed videos to enable the detection of tics that span multiple input video segments. However, the analysis was conducted on data from a single patient, limiting the generalisability of the findings. In contrast, our study evaluates performance across multiple patients in a subject-independent setting, making direct comparisons challenging. Wu et al. [42] developed a tic detection algorithm based on visual features, demonstrating the efficacy of pre-training a neural network on unlabelled image data to enhance results when labelled videos are scarce. They used an unsupervised contrastive learning network on 2D images and trained a supervised LSTM classifier on the extracted representations. In the study of Brügge et al. [10], two supervised automated tic detection methods were developed, namely a random forest classifier and a 3D deep neural network. The binary classification of one-second windows into tic and non-tic categories was accomplished using a relatively large dataset of videos of GTS patients and fine-grained annotations. Another study by Brügge et al. [43] incorporated privacy preservation into tic detection by pre-training a feature model on public data and using a downstream classification model, significantly reducing the success of privacy attacks.

In contrast to all aforementioned studies, our current work addresses a more challenging scenario, as we assume that we do not have access to per-second labels in training, which is often the case in medical study datasets. We leverage an even larger dataset than the one presented in [10] and only weakly-labelled videos. We therefore propose a versatile top-*k* MIL approach applicable to a wide range of medical tasks involving facial video data. As in [12], it allows for capturing temporal patterns within longer video sequences.

### Multiple Instance Learning in Medical Image and Video Analysis

MIL has shown promising results in medical image analysis tasks, for instance in the domain of brain MRI analysis for the classification of dementia [44], in color fundus images for diabetic retinopathy detection [45], and bone scintigraphy [46]. Multiple works have applied MIL to histology patches in cancer research. Li et al. [47] used MIL with weakly correlated histopathological images and clinical information to predict lymph node metastasis in breast cancer. Kandemir et al. [45] classified esophagus cancer using MIL. Xu et al. [48, 49] utilised MIL for analysing colon cancer histopathology patches.

For medical videos, Sikka et al. [50] proposed weakly-supervised pain localisation using MIL for pain detection in medical videos. Similarly, MIL has been applied to bag-level stress detection from facial video data to identify stress-related expressions [51, 52] without requiring frame-level annotations. Contrastive transformer-based MIL was applied to weakly-supervised polyp frame detection in colonoscopy videos [53]. These works demonstrate the versatility and effectiveness of MIL in various medical image and video analysis tasks.

## Dataset

We used data from two different studies, the first one being similar to the data used in past studies such as [10, 54–56] and the second one being a Rush tic study [57]. This data is described in more detail in the following two sections and in Appendix A.

Patients with GTS were recruited through an outpatient clinic at the Neurology Department of the University Hospital Schleswig-Holstein, Campus Lübeck, Germany. The diagnosis of GTS was based on the DSM-5 criteria [34]. Exclusion criteria for the study include an IQ below 80, recent medication changes within the previous four weeks, and a history of significant psychiatric or neurological disorders other than Obsessive-Compulsive Disorder or Attention-Deficit/Hyperactivity Disorder. Written consent was obtained from all participants, and the study received ethics board approval from the universities of Lübeck and Dresden (EK 359092017, Aktenzeichen: 17-156).

### Densely Labelled GTS Video Dataset

For the evaluation of our approach, we used a second-by-second labelled dataset. Data acquisition followed the protocol described in [55]. The dataset comprises 64 videos of 35 individuals, including 42 videos of children and 22 videos of adults with GTS. The mean ages in the children and adult groups were *12.6± 2.4* years and *30.9± 10.6* years, respectively. The video frame captures each participant’s upper body and face.

Two independent clinical raters rated each of the 5-minute videos per second regarding the presence and the severity of tics. They were previously trained by an expert. All segments where the two raters disagreed regarding the presence or absence of a tic were reviewed again by a third expert (A.M.), who made the final decision. Tic intensity was assessed using a clinical rating scale that was also used in [54, 55] (see Table 3 in the Appendix).

### Weakly-Labelled GTS Video Dataset

We use Rush video recordings as large weakly-labelled dataset. In total, we use 345 videos for training, including 160 videos showing GTS patients and 185 videos showing HC subjects. These recordings are of 10 min duration and consist of *2 ×* 2.5 min showing a whole body view and *2 ×* 2.5 min focusing on the area of the head and shoulders. Subjects were seated in front of the camera.

**Fig. 2:**
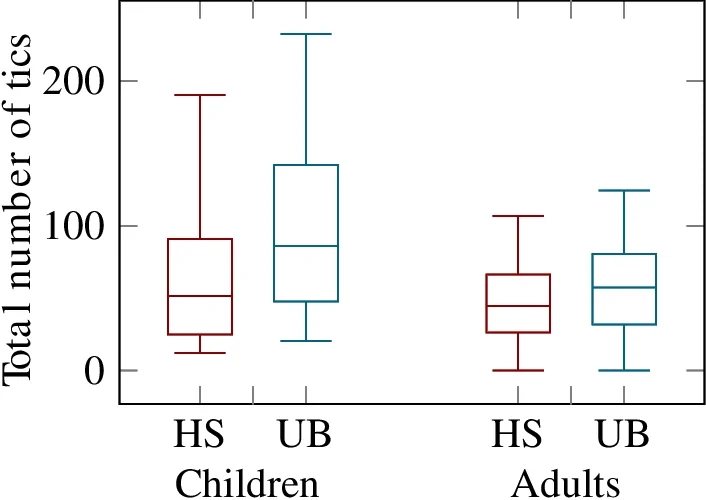
Boxplot showing the distribution of the number of head and shoulder tics (HS) and upper body tics (UB) per 2.5 min for children and adult patients in the weakly-labelled training dataset

We exclusively used the close-up part of the video to ensure adequate resolution of the facial area and enable robust face and landmark detection. The videos were not labelled second by second, but were given labels per video that included the number of head and shoulder tics per minute, motor tic severity, and final Rush scores [57]. We use as annotations the number of head and shoulder tics $s_{i,\text {head/shoulder}}$ and the total number of upper body tics $s_{i,\text {upper}}$, where *i* designates the video index. The latter also includes the number of blinks minus 43, following the assumption that a healthy person blinks 43 times per 2.5 minutes on average while resting [58]. The score values $s_{i,\text {head/shoulder}}$ have a mean value of 53.83, a standard deviation of 39.19, and range between 0 and 207. The scores $s_{i,\text {upper}}$ have a mean value of 61.80, a standard deviation of 43.91, and lie in the range between 0 and 216. A boxplot showing the distribution of both scores is shown in Fig. 2.

## Methods

We organise our methodology into two key sections, addressing contrastive learning feature extraction and introducing the proposed modification to MIL within the context of our study.

### Contrastive Learning Feature Extraction

Typically, MIL models rely on off-the-shelf feature networks, such as C3D or I3D, which are trained on action recognition datasets like Kinetics-400 [59]. These networks are well-suited for detecting anomalies in street scenes, aligning with the baseline model’s intended purpose. However, these networks may not yield highly descriptive features for detecting medical anomalies in facial video data due to the significant dissimilarity between the datasets used for their training and the target medical application.

In light of this limitation, we investigate a feature extraction approach specifically tailored to our medical anomaly detection task to improve the performance of anomaly detection in facial video data. As a customised approach for facial video data, we use a contrastive learning model based on SimCLR [60] which was trained on a large unlabelled public dataset containing videos of persons recorded for emotion recognition [61]. As the backbone model, we use a 3D ResNet-18 [29]. Previous work [43] demonstrated that this architecture can extract features that are discriminative for medical video analysis tasks, despite being trained exclusively on data from healthy individuals.

The approach involves the extraction and tracking of facial landmarks. Therefore, it is necessary to use tailored transformations for contrastive learning, such as horizontal landmark flipping as well as global and local scaling, which is implemented by multiplying *x*- and *y*-coordinates by random factors. These adaptations are applied to both the complete set of landmarks and individual face parts like eyes or mouth, leading to invariance to the inherent diversity in facial shapes and characteristics. To capture facial expressions and head movements, the same transformation is applied to all frames in one model input sequence.

### Multiple Instance Learning

#### Motivation

In MIL, the learning paradigm aims to leverage weak labels at the bag level to infer unknown instance-level labels within each bag. Top-*k* MIL [14, 62, 63] addresses the task of identifying the top-*k* instances within each bag that are most likely to be positive examples and learning the correct instance-level classification based on this information. The data is divided into positive and negative bags, where positive bags contain at least one positive sample and negative bags contain no positive samples. Previous work has shown that the separation of positive and negative instances works particularly well when *k* is approximately equal to the number of positive instances in a positive bag [63].

In the context of tic detection from videos, positive samples represent seconds showing tics and negative samples seconds that do not contain any tic movements. In the case of tic detection in Tourette patients, the number of positives within positive bags can vary significantly since the severity of the disorder varies among patients (see Fig. 2). In these cases, determining a suitable *k* is challenging: a small *k* minimises false positive snippets but risks discarding relevant information (see bag C in Fig. 1), while a large *k* includes an excess of normal snippets, hindering robust classifier training (see bag A in Fig. 1). Setting *k* to a constant value may therefore not be optimal. The latter could result in only the snippets showing strong tics being used to train the classifier and weaker tics not being detected. We adopt an adaptive approach by dynamically selecting a suitable value of *k* based on the characteristics of individual bags.

#### Score-Driven Top-k Multiple Instance Learning

**Fig. 3:**
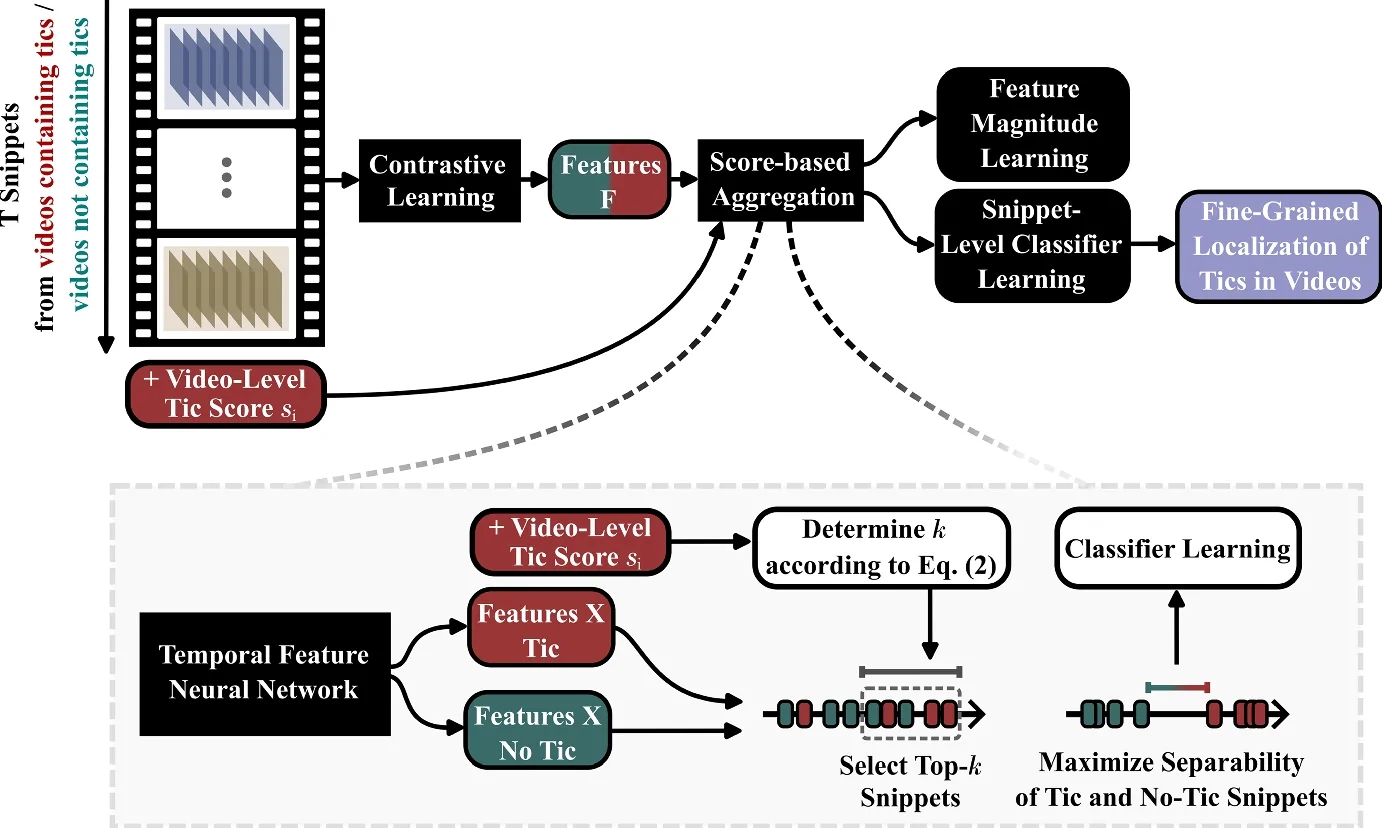
Overview over the proposed multiple instance learning approach for tic detection. First, the video is cut into *T* snippets of 1 s. Second, features are extracted using the contrastive learning feature network pre-trained on unlabelled video data. The features are input to a neural network capturing temporal patterns across multiple snippet features

Our general approach is based on [14], as this is one of the state-of-the-art approaches and can be adapted well to our problem. Here, the assumption is made that each positive bag in the dataset contains approximately *μ* positive snippets. This assumption usually does not hold in medical video analysis. Therefore, we assume that each positive bag can contain a significantly different number $\mu _i$ of positive snippets, where a positive bag corresponds to a video showing a person with GTS. In this work, we aim to estimate $k_i$ in top-*k* MIL for each video *i,* so that $k_i \approx \mu _i$, to make the best use of the available training data. An overview of the approach can be seen in Fig. 3.

In our proposed framework, each bag corresponds to a set of *T* snippets of fixed length *L* seconds, randomly extracted from the video. From each snippet, a *D* dimensional feature vector is extracted using the pre-trained contrastive learning feature network. Each video *i* is therefore represented by a set of features $\textbf{F}_i \in \mathbb {R}^{T \times D}$. We denote the training dataset of weakly-labelled Rush tic recordings by $\mathcal {D}=(\textbf{F}_i, {y_i, \tilde{y}}_i)^{|\mathcal {D}|}_{i=1}$, where $y_i$ is the binary label whether the video shows a HC subject ($y_i=0$) or a subject with GTS ($y_i=1$) and $\tilde{y}_i$ is the corresponding weak video-level tic score label. The score label $\tilde{y}_i$ correlates with the tic frequency of the corresponding patient and takes the value $\tilde{y}_i=y_i=0$ if the video shows a HC person and $\tilde{y}_i>0$ if the person in the video was diagnosed with GTS. We define $\tilde{y}_i$ for the *i*-th video as

1 $$\begin{aligned} \tilde{y}_i=\frac{s_i}{\tau _i}, \end{aligned}$$

where $s_i$ is the number of tics in video *i*, and $\tau _i$ its length in seconds. Multiplying $\tilde{y}_i$ by *T· L* gives an estimation of the average total number of tics contained in one bag. Therefore, we select $k_i$ based on this tic score label by

2 $$\begin{aligned} k_i = \min (T, \max (c_{\text {cutoff}}, \tilde{y}_i \cdot T \cdot L \cdot c_{\text {bout}})). \end{aligned}$$

We introduce a fixed correction factor $c_{\text {bout}}$ to account for the likelihood of multiple tics occurring within one second, as they tend to appear in bouts and choose a lower bound for $k_i$ of $c_{\text {cutoff}}$. Additionally, if $\tilde{y}_i \cdot T \cdot L \cdot c_{\text {bout}}> T$, we set $k_i=T$.

Following the approach in [14], we employ the MTN temporal feature network $s_\theta :\mathcal {F}\xrightarrow {}\mathcal {X}$ for the extraction of temporal features $\textbf{X} = s_\theta (\textbf{F})$ from the features $\textbf{F}$. We did not modify this network since it is already well suited for the analysis of our data. The architecture consists of two branches: one that extracts multi-scale temporal features using pyramid dilated convolutions and another that incorporates global temporal relations via self-attention. Based on the temporal features, a snippet-level classification network $f_\phi :\mathcal {X}\xrightarrow {}[0,1]^T$ generates the binary *T*-dimensional classification of whether a video contains tics by $f_\phi (s_\theta (\textbf{F}))$.

Similar to [14], we denote features of positive and negative snippets as $\textbf{x}^+ \sim P^+_x(\textbf{x})$ and $\textbf{x}^- \sim P^-_x(\textbf{x})$, where a positive video $\textbf{X}^{+}$ showing tics can contain snippets drawn from both $P^+_x(\textbf{x})$ and $P^-_x(\textbf{x})$ but negative videos $\textbf{X}^{-}$ showing HC subjects can only contain snippets from $P^-_x(\textbf{x})$. We also make the assumption that ${\mathop {\mathbb {E}}} \left[ \Vert \textbf{x}^+ \Vert _2\right] \ge \mathop {\mathbb {E}} \left[ \Vert \textbf{x}^- \Vert _2\right]$ where $\textbf{x}^+$ and $\textbf{x}^-$ denote single snippet features showing tics and no tics respectively. This means that it is possible to train the network $s_\theta$ to produce non-tic snippet features $\textbf{x}^{-}$ with smaller magnitude than that of tic snippet features. A snippet feature $\textbf{x}_t$ represents the *t*-th row in $\textbf{X}$ with *t=1,...,T*.

The loss for the joint training of the classifier $f_\phi$ and the temporal feature network $s_\theta$ is given by

3 $$\begin{aligned} {\begin{matrix} \ell _{\text {overall}} = \min _{\phi , \theta } \sum \limits ^{|\mathcal {D}|}_{i,j=1} \sum \limits ^{N_i}_{n=1} & \ell _s\left( s_\theta (\textbf{F}^{(n)}_i), s_\theta (\textbf{F}^{(n)}_j), y_i, y_j\right) \\ + & \ell _f \left( f_\phi \left( s_\theta (\textbf{F}^{(n)}_i)\right) , y_i\right) , \end{matrix}} \end{aligned}$$

where $N_i$ is the number of input sub-videos of length *T* that are extracted from one recording. We assume that $\mu _i$ is approximately the same for all $N_i$ sub-videos of one recording and therefore use the same $k_i$ for all $\textbf{X}^{(n)}$. The loss function $\ell _{\text {overall}}$ combines a snippet classification loss $\ell _f$ being the standard cross-entropy loss, and a feature separability loss function $\ell _s$ maximising the separability between top-$k_i$ features of HC and GTS videos.

For the feature separability loss $\ell _s$, we adopt the optimisation scheme from [14] and adapt it to the use of different $k_i$ for each GTS video. For this, we determine the top-$k_i$ snippet features from the *i*-th video and calculate the mean feature norm by

4 $$\begin{aligned} g_{\theta , k_i}(\textbf{X}) = \max _{\Omega _{k_i}(\textbf{X})\subseteq \{\textbf{x}_t\}^T_{t=1}} \frac{1}{k_i} \sum _{\textbf{x}_t\in \Omega _{k_i}(\mathbf {X)}}\Vert \textbf{x}_t \Vert _2 \end{aligned}$$

where $\Omega _{k_i}(\textbf{X})$ is a subset of $k_i$ snippets in $\{ \textbf{x}_t \}_{t=1}^T$. With a pre-selected margin *m*, we note the separability loss as

5 $$\begin{aligned} {\begin{matrix} &\ell _s \left( s_\theta (\textbf{F}_i), s_\theta (\textbf{F}_i), y_i, y_j\right) = \\ & {\left\{ \begin{array}{ll} (|m-g_{\theta , k_i}(\textbf{X}^+)| + g_{\theta , k_i}(\textbf{X}^-))^2 & \text {if } y_i=1, y_j=0, \\ 0 & \text {otherwise} \end{array}\right. } \end{matrix}} \end{aligned}$$

where we use the same $k_i$ determined based on the GTS video $\textbf{X}^{+}$ for both $\textbf{X}^{+}$ and $\textbf{X}^{-}$. This ensures that we use a balanced number of GTS and HC snippets for training.

The classification loss $\ell_f$ is given by

6 $$\begin{aligned} {\begin{matrix} &\ell_f \left( f_{\phi }\left( s_{\theta }(\textbf{F})\right) , y\right) = \\ & \sum _{\textbf{x} \in \Omega _{k_i, \max }(\textbf{X})} -\left( y \log \left( f_{\phi }(\textbf{x})\right) + (1 - y) \log \left( 1 - f_{\phi }(\textbf{x})\right) \right) . \end{matrix}} \end{aligned}$$

This classification loss is also getting the top-$k_i$ features from $\textbf{X}_{i}$ with the largest L2 norm as input.

## Experiments

Following the experimental setup of [10], we considered a binary classification task between tics and no tics. For the evaluation of the snippet-level predictions, we used the second-wise labelled data, described in Section 4.1 . A second was annotated as part of the tic class if its corresponding tic intensity label was greater than or equal to a threshold of three (see Table 3 in the Appendix for details). All videos were resampled to a frame rate of 30 frames per second to match the labelled video recordings. Subsequently, we cut the video into non-overlapping snippets of 1 s, each containing 30 frames. As a first step in MIL, it is important to extract informative features from these video snippets. Similar to the baseline model from [14], we extracted features using the Inflated 3D ConvNet (I3D) [30] and compared these features with those obtained from the proposed contrastive learning approach.

### I3D Feature Network

The I3D feature network is a widely used video feature extractor, pre-trained on the large-scale Kinetics-400 dataset [59]. It extends 2D convolutional neural networks into the temporal domain by inflating 2D filters into 3D, enabling it to jointly capture spatial and temporal information. The architecture consists of two separate streams: the *RGB stream* capturing visual content and the *optical flow stream* modelling motion by analysing frame-to-frame displacement patterns. This fusion makes I3D features effective for tasks like action recognition and video analysis, as they encapsulate appearance and motion cues within video sequences. For the I3D model, the feature dimension is *D=4096* (2048 for each stream).

### Contrastive Learning Feature Network

As a contrastive learning feature extraction model, we use a landmark-based model, which was pre-trained on facial video data of healthy subjects. The network architecture is outlined in Section 5.1 and Appendix B and illustrated in Fig. 7. For the application of this model, we additionally tracked 478 facial landmarks using Google’s mediapipe [64]. The output dimension of the resulting features is *D=512*.

### Model Training and Evaluation

As a validation set for hyperparameter tuning, we used the data of eight subjects. These subjects were excluded from the training and testing sets, which consist of the remaining weakly and densely labelled videos, respectively. The number of features per bag was set to *T=50*, with features being extracted from video snippets of *L=1* second duration. To calculate $k_i$ (see (2)), we set $c_{\text {bout}} = 0.8$ and $c_{\text {cutoff}} = 5$, based on the assumption that each video contains a non-zero number of tics, aligning with the principle of MIL. The value of $c_{\text {bout}}$ was selected using the validation dataset. All models were trained for 100 epochs, using Adam optimisation with a learning rate of 0.0005 and a batch size of 32. The learning rate was multiplied by a factor of 0.9 every 5 epochs. We set the margin *m* in the separability loss to 100. We repeated the model training process ten times, each time with a different seed, while using the same seeds across all models. To ascertain the optimal performance of the baseline models, we conducted tests with different fixed values of *k*.

Similar to prior work on MIL [14, 20–24], we used the Area Under the Curve (AUC) as metric, as it quantifies the model’s ability to discriminate between positive and negative instances across different classification thresholds. The reported AUC values are averaged over all runs.

### Baseline Models

We additionally evaluated our MIL tic detection approach against other MIL models that achieved state-of-the-art results on a variety of surveillance camera anomaly detection datasets [14–19]:

- *UR-DMU* [17] uses Uncertainty Regulated Dual Memory Units to learn distinct representations for both normal and abnormal events in weakly supervised video anomaly detection. To capture video associations, they use global and local multi-head self-attention modules within a transformer network.
- *S3R* [16] integrates dictionary learning with self-supervised techniques to capture feature-level anomalies. A dictionary is created by learning sparse representations, where each feature is represented as a linear combination of basic elements, or atoms. Using this dictionary, S3R employs two coupled modules, en-Normal and de-Normal, to reconstruct snippet-level features and filter out normal features. We use the video data of the healthy subjects to learn a dictionary. To solve the lasso problem, we use the least angle regression method.
- *BN-WVAD* [19] addresses the challenge of temporal ambiguity in weakly supervised video anomaly detection by integrating BatchNorm. BN-WVAD uses the divergence of features from the mean of BatchNorm as an additional anomaly criterion to help identify abnormal snippets. Similar to our approach, the key idea is to dynamically identify and extract more abnormal snippets from videos with a higher incidence of abnormal events, allowing for a more effective supervision for anomaly detection.
- *WSAL* [15] employs a high-order context encoding model to generate anomaly scores that also incorporate dynamic variations and are aggregated using a margin loss function.
- *Contrastive Transformer-based Multiple Instance Learning for Weakly Supervised Polyp Frame Detection* [14] comprises a joint optimisation of a transformer-based temporal feature learning, a bag-level classifier and contrastive snippet mining for training the MIL classifier.
- *MGFN* [18] introduces a Glance and Focus Network for weakly-supervised video anomaly detection, which integrates spatial and temporal information. To overcome inconsistencies in feature magnitudes across varying scenes, a feature amplification mechanism and magnitude contrastive loss are employed.

## Results

### Feature Extraction

**Fig. 4:**
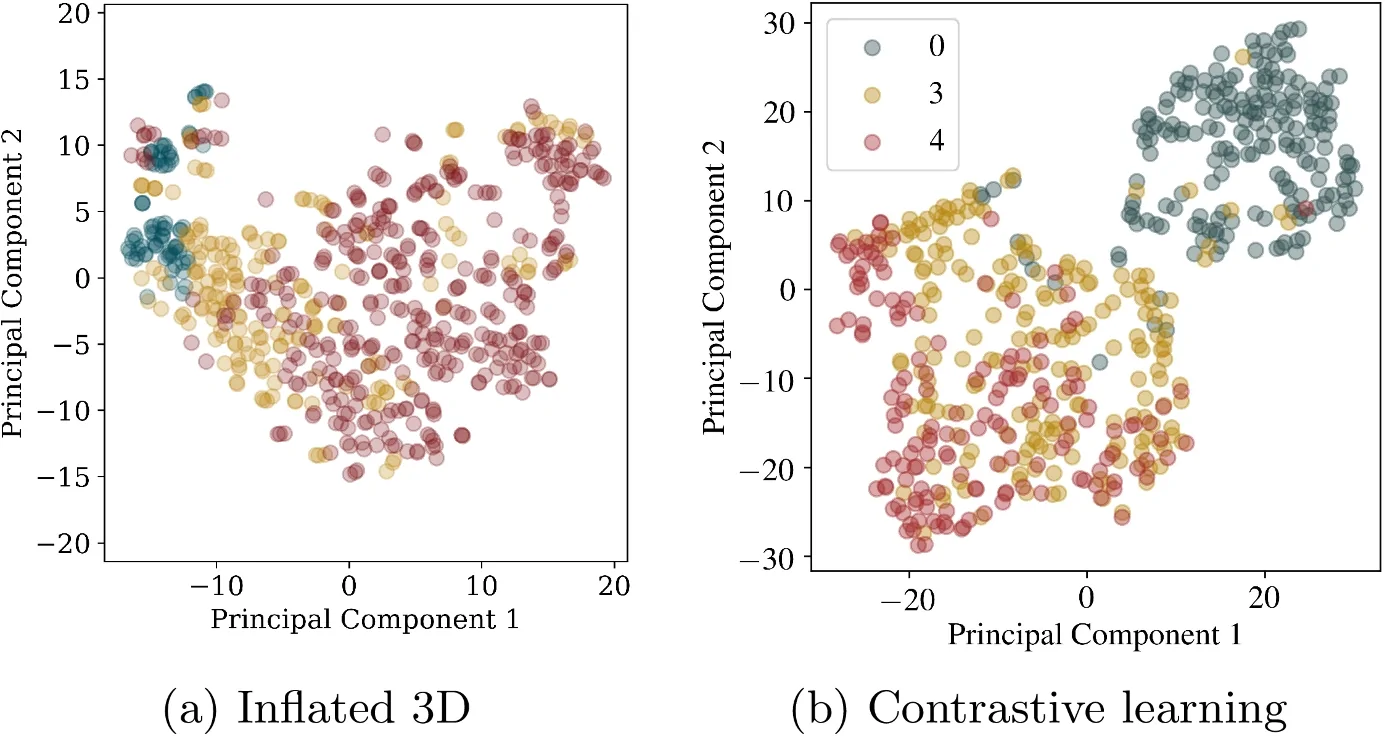
T-SNE visualisation of the features extracted using either (**a**) the I3D network pre-trained on Kinetics-400 [59] or (**b**) the contrastive learning feature network pre-trained on facial video data from [61]. The values in the legend represent the tic severity

We projected the extracted features by both the I3D and the contrastive learning models onto their first two principal components using t-SNE. Figure 4 shows the plot for the features of the same patient. In the I3D feature plot, there is a larger overlap of snippets with tic severity ranging from 0 to 4 (see Table 3 for more details on the severity rating). In contrast, the feature representation learned by the contrastive learning model yields more distinct clusters of tic and non-tic video segments. Notably, subtle tics rated with severity 3 are positioned closer to the non-tic cluster, whereas more pronounced tics (severity 4) are more clearly separated, reflecting a gradation of tic intensity in the learned embedding.

### Snippet-Level Classification

**Table 1:** AUC values for the RTFM baseline model for different values of *k* and for the proposed score-based top-*k* MIL model. Values are each averaged over 10 runs. I3D and CL represent the feature extraction method with either an Inflated 3D Convolutional network (I3D) or contrastive learning (CL)

	RTFM baseline model with fixed values of *k*											Variable *k*
*k=*	1	3	5	7	10	15	20	25	30	35	40	
I3D	74.88	73.63	72.61	72.46	71.46	71.48	71.46	71.27	71.06	71.03	70.70	$\mathbf {75.19}$
CL	79.52	85.86	86.09	85.83	85.46	84.10	83.77	84.25	84.23	84.14	84.32	$\mathbf {86.73}$

**Fig. 5:**
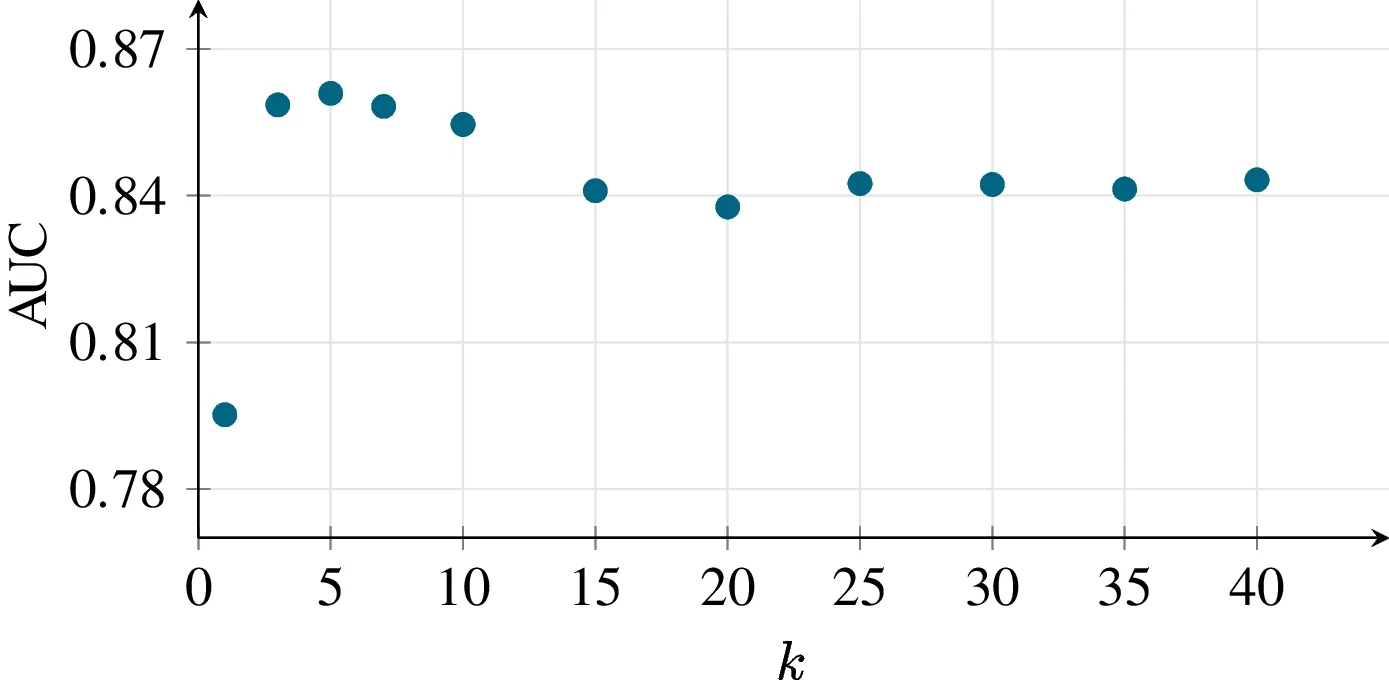
AUC values obtained for different fixed values of *k* in the baseline model using contrastive learning features

Table 1 shows the AUC values obtained for the snippet-level tic classification based on either the I3D or contrastive learning features using the baseline framework from [14]. We list the AUC for different values of the hyperparameter *k* in the RTFM baseline model and for the adapted model with varying hyperparameter $k_i$ per video *i*. As Fig. 4 suggests, the contrastive learning features led to superior results compared to the I3D features. For the RTFM baseline model with a fixed *k*, the table shows that selecting a value of *k=5* yields the highest AUC of *86.09 %*. Figure 5 also illustrates that the classification performance peaks at *k=5* and decreases with lower and higher values of *k*.

We obtain an even higher AUC of 86.73 % with a dynamically adapted value of $k_i$, depending on the tic score $s_i$ of each individual video *i*. To test the significance of the difference in the results, we first performed 10 more runs with different seeds in both the RTFM baseline with *k=5* and the adaptive approach, as this increases the significance of the following statistical tests. To test for normality on the differences of these results, we performed a Shapiro-Wilk test. It showed that there was no significant deviation from normality (*W(20)=0.9848*, *p=0.9808*). A paired sample t-test showed that the difference between the two models is significant (*t(19)=5.5037,~p<0.001*). It can therefore be said that the score-driven model performs better than the fixed-*k* baseline.

**Table 2:** Tic detection AUC values for different MIL models trained and evaluated on the contrastive learning features

Model	Tic Detection AUC (%)
UR-DMU [17]	78.98
S3R [16]	80.34
BN-WVAD [19]	80.94
WSAL [15]	81.87
Transformer-based [53]	84.56
MGFN [18]	84.73
RTFM [14]	86.09
Ours	**86.73**

Table 2 shows the AUC values of the baseline models on our tic detection dataset. As in the previous experiments, we used the contrastive learning features described in Section 5.1. Unlike prior work on MIL, we do not report the best AUC value obtained during training. Instead, we optimised the model hyperparameters on a validation subset and report the AUC after the model has been trained, as this provides more representative results for practical use. On the tic detection dataset, we found that our adapted model performed best with an AUC of *86.73 %*, followed by RTFM, MGFN [18] and a transformer-based approach that was originally developed for colon polyp detection [53], achieving tic detection AUCs of *86.09 %*, *84.73 %* and *84.56 %*, respectively.

**Fig. 6:**
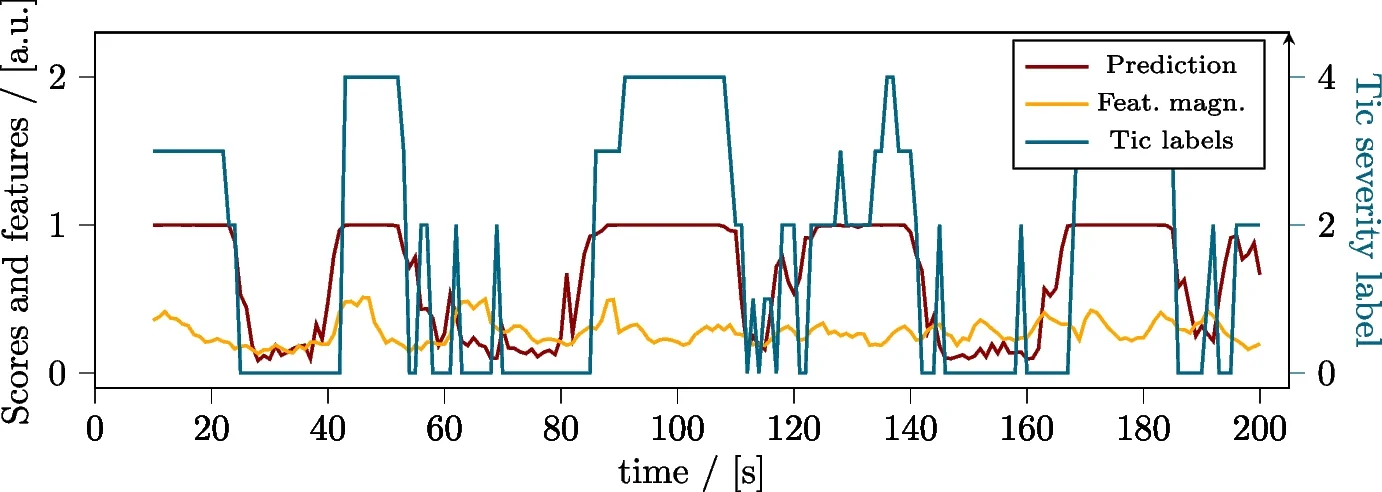
Model prediction (Prediction), temporal network feature magnitudes (Feat. magn.) and ground truth tic labels (Tic labels) of an exemplary GTS video within a time window of 200 s obtained with the proposed top-*k* MIL approach based on contrastive learning features

**Fig. 7:**
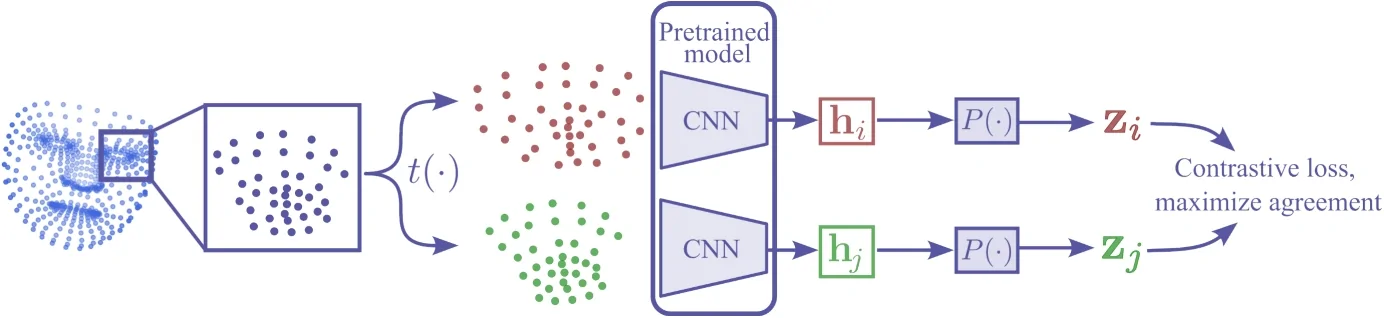
Contrastive Learning Model used for unsupervised feature representation learning on landmarks. Similar to [43], two augmentations of facial landmarks from a video segment were input to a 3D ResNet-18. Through the projection head *P(· )*, the representations $h_i$ and $h_j$ are projected into the space where the contrastive loss is applied

Figure 6 shows the predictions of the classification network and the feature magnitudes of the temporal feature network of the adaptive model together with the ground truth tic severity labels within a time window of 200 s. It is apparent that even though the model was trained without second-wise supervision, there is a strong correlation between the predicted scores and the tic labels over time.

## Discussion

This section discusses the results of tic detection, highlights key findings, states the limitations of the study and identifies avenues for further investigation.

### Choosing the Value for *k*

We could show that choosing a variable parameter *k* for MIL yields superior results compared to fixing it to a specific value, as done in previous work. In [63], it could be shown that MIL leads to the best results if the value of *k* is approximately equal to the number of events to be detected. In our study, this would suggest that there is an average number of 5 tics in one sequence of video snippets used for training the model. Therefore, when averaging all per-video $k_i$, one would expect an average *k* that is close to the fixed-*k* optimum *k=5*. However, in this setting we observe a mean *k* value of 14.91 with a high standard deviation of 11.87, though Fig. 5 implies that a fixed *k≈ 15* is not optimal. This indicates that adjusting *k* based on background knowledge is useful for MIL. A possible explanation for this is that the limited amount of training data can be better utilised without including many false positive instances into the training process, as illustrated in Fig. 1.

### Extend to Second-by-second Classification

The classifier network predictions, the temporal feature network sizes, and the ground truth tic severity show a strong correlation, indicating that the model performs well even without second-wise supervision during training, as shown in Fig. 6. By determining a suitable threshold for the classifier output, it would be possible to accurately identify time segments in which tics occur. For this, a smaller, densely-labelled sub-dataset could be used, for example, to train a simple linear model to predict the optimal threshold.

### Comparison to Other Methods

A direct comparison of the approach with established baselines on public datasets proves challenging due to the absence of weak labels, which are essential for quantifying anomalous events. Additionally, the contrastive learning approach is specifically tailored to facial video data and may not generalise well to other domains, such as surveillance camera videos. We therefore compared our adapted approach to different state-of-the-art MIL models for anomaly detection on our medical dataset. The proposed adapted model achieved the best performance, followed by RTFM and MGFN, despite MFGN demonstrating superior performance on the surveillance camera datasets [18]. MGFN includes a contrastive loss that improves performance on heterogeneous datasets with different backgrounds and detected objects. However, this improvement was not observed for the tic detection dataset. This discrepancy may be attributed to two key factors: firstly, all recordings were conducted within a single, controlled environment, and secondly, contrastive learning based on facial landmarks reduces the impact of varying recording conditions, including lighting differences due to different times of day, distances between the test subject and the camera, and background variations.

The comparison with existing tic detection methods is difficult due to the use of significantly differing input data, additional sensors, dense labels, and non-public medical datasets. One advantage of our approach over supervised learning lies in its independence from the correctness and consistency of clinical tic ratings, which allows for the use of large-scale unlabelled datasets from clinical practice.

### Limitations and Practical Considerations

A potential limitation of this study arises when dividing videos into shorter segments, as it cannot be guaranteed that the mean tic count per minute remains precisely consistent across each sub-video. This variance may result in slight deviations in the tic counts per minute used to calculate the weak score labels.

While our feature extraction is based on non-overlapping 1 s video snippets, we acknowledge that longer or overlapping snippets could potentially offer additional temporal context. We also note that in this work, the snippet length was fixed across all experiments and was not treated as a tunable hyperparameter. Future research could explore the impact of varying this length on model performance. However, the MIL approach incorporates global attention across all video snippets within a segment, allowing the model to reason over broader temporal dependencies.

It remains to be investigated how the algorithm performs on datasets that are not obtained based on either of the two study protocols. However, since contrastive learning makes the inputs invariant to many external influences, it is expected that the approach will also perform well on diverse other datasets. Yet, it should be ensured that the input video matches the training data as closely as possible. This is particularly important with respect to the frame rate, as different frame rates have not been integrated into the contrastive learning process in order to not remove tic-related information.

## Conclusion

In this study, we demonstrated the efficacy of weakly-supervised MIL for medical facial video analysis. Specifically, it was possible to localise tics in videos showing subjects with GTS by using only weak video-level labels.

For feature extraction, we used a pre-trained contrastive learning network trained to focus on capturing facial movements and expressions. As the MIL aggregator, we modified a top-*k* MIL approach based on [14]. To quantify tic occurrences in each video, we used video-level labels that were obtained within the context of Rush tic labelling. This information was used to determine a suitable parameter $k_i$ for each video *i* in top-*k* MIL. The motivation was to address the substantial variation in tic frequency between patients. Our approach outperformed the baseline models, achieving the highest area under the curve at 86.73 %, highlighting the efficacy of leveraging weakly-labelled data. Using MIL in medical video analysis is a natural approach given the large amount of unlabelled video that can be easily recorded and the time-consuming process of manual annotation that requires the expertise of medical professionals. Additionally, medical data are often tagged with additional metadata that can provide information about the number of abnormal instances. Our proposed extension is especially useful in these datasets. In future work, we aim to investigate semi-supervised approaches to additionally exploit the potential of fully annotated videos.

## Data Availability

The video data are not openly available due to reasons of sensitivity and privacy of the study participants. Data that support the findings of this study are available from the corresponding author upon reasonable request.

## Appendix A: Data Acquisition and Clinical Assessment

As the labelled evaluation dataset, we used a study dataset from a tic urge study similar to [54–56]. In this study, the patients were seated in front of a computer screen displaying an urge scale. They moved a cursor to rate their urge to tic. The frames covered the head, face, upper body, and hands at a distance of 60 cm from the camera. We used room lighting and daylight from a window. Data were collected for 5 min. Motor tics were rated using a tic intensity rating scale shown in Table 3.

In the Rush study, only video-level annotations were given, which included the Rush scores describing the motor tic and vocal tic frequency and severity, and total tic counts (shoulder, head, blinking, whole body and upper body). In this study, the patients did not perform a given task but were seated in front of a camera for 10 min.

Video and audio were captured with Logitech c930 webcams at 30 Hz in the Urge tic study and 25 Hz in the Rush study. The resolution of the videos was *1920 × 1080* pixels.

A clinical assessment was conducted as described in a publication from [55]. The severity of Gilles de la Tourette syndrome symptoms was rated by the Yale Global Tic Severity Scale [65] and Diagnostic Confidence Index [66]. To detect obsessive compulsive disorder, the Yale Brown Obsessive Compulsive Scale [67] and the Children’s Yale Brown Obsessive Compulsive Scale [68] were assessed for adults and children, respectively. ADHD was evaluated by using the Conners Child ADHD Rating Scale for children [69] and the Conners Adult ADHD Rating Scale for adults [70]. The Premonitory Urge for Tics Scale [71] was used for indicating premonitory urges.

**Table 3:** Clinical tic rating scale for motor tics

Rating	Description	Example
0	None	
1	very mild, could be a normal movement	short sniff
2	mild, clearly tic; undoubtedly noticeable to tic experts but may not be noticed by others	nasal flare; opening eyes
3	mild; clearly a tic, noticeable to any observer attending to it, no exaggerated muscle activity	mouth pouting, kiss
4	moderate, easily noticeable, exaggerated muscle activity	raising the nose, screwing up the eyes
5	moderate to severe, exaggerated muscle activity leading to unmissable distortion	tension throughout the whole body, head to the side for a longer period
6	A severe, unusual movement clearly raises attention	staring, cramped fist knocking on the table
7	A severe, unusual movement that is off-putting or frightening	mouth wide open and cramped middle finger pointing
8	very severe, i.e., apparently painful or potentially dangerous e.g., due to self-injury	hitting in the face

## Appendix B: Contrastive Learning Model

Contrastive learning is a self-supervised learning technique used to train neural networks by maximising the similarity between augmented views of the same data sample while minimising the similarity between views from different samples.

In our approach, we used a 3D ResNet-18 backbone with facial landmarks as input. Specifically, the input consists of a set of *30× 478* landmarks for each 1 s video input with a frame rate of 30 fps. The landmarks were tracked using Google’s Mediapipe [64] as in [43]. To ensure compatibility of the model with the landmark input, we used adapted transformations, including global and local scaling or shifting of the coordinates. These transformations are applied equally to each set of facial landmarks in one video segment to ensure temporal consistency. The use of landmarks in conjunction with contrastive learning should make the input invariant to different lighting conditions, face shapes and backgrounds. An overview of the feature network is given in Fig. 7. This contrastive learning framework allowed us to learn relevant features from the facial video data, enhancing the performance of our model for detecting tics.
